# Origin and evolutionary history of domestic chickens inferred from a large population study of Thai red junglefowl and indigenous chickens

**DOI:** 10.1038/s41598-021-81589-7

**Published:** 2021-01-21

**Authors:** Ayano Hata, Mitsuo Nunome, Thanathip Suwanasopee, Prateep Duengkae, Soontorn Chaiwatana, Wiyada Chamchumroon, Takayuki Suzuki, Skorn Koonawootrittriron, Yoichi Matsuda, Kornsorn Srikulnath

**Affiliations:** 1 Laboratory of Avian Bioscience, Department of Animal Sciences, Graduate School of Bioagricultural Sciences, Nagoya University, Nagoya, Aichi 464-8601 Japan; 2 Laboratory of Animal Cytogenetics and Comparative Genomics (ACCG), Department of Genetics, Faculty of Science, Kasetsart University, Chatuchak, Bangkok, 10900 Thailand; 3 Tropical Animal Genetic Unit (TAGU), Department of Animal Science, Faculty of Agriculture, Kasetsart University, Chatuchak, Bangkok, 10900 Thailand; 4 Avian Bioscience Research Center, Graduate School of Bioagricultural Sciences, Nagoya University, Nagoya, Aichi 464-8601 Japan; 5 Special Research Unit for Wildlife Genomics, Department of Forest Biology, Faculty of Forestry, Kasetsart University, Chatuchak, Bangkok, 10900 Thailand; 6 Department of National Parks, Wildlife and Plant Conservation, Chatuchak, Bangkok, 10900 Thailand

**Keywords:** Evolutionary biology, Population genetics, Biodiversity, Conservation biology

## Abstract

In this study, we aimed to elucidate the origin of domestic chickens and their evolutionary history over the course of their domestication. We conducted a large-scale genetic study using mitochondrial DNA D-loop sequences and 28 microsatellite DNA markers to investigate the diversity of 298 wild progenitor red junglefowl (*Gallus gallus*) across two subspecies (*G. g. gallus* and *G. g. spadiceus*) from 12 populations and 138 chickens from 10 chicken breeds indigenous to Thailand. Twenty-nine D-loop sequence haplotypes were newly identified: 14 and 17 for Thai indigenous chickens and red junglefowl, respectively. Bayesian clustering analysis with microsatellite markers also revealed high genetic diversity in the red junglefowl populations. These results suggest that the ancestral populations of Thai indigenous chickens were large, and that a part of the red junglefowl population gene pool was not involved in the domestication process. In addition, some haplogroups that are distributed in other countries of Southeast Asia were not observed in either the red junglefowls or the indigenous chickens examined in the present study, suggesting that chicken domestication occurred independently across multiple regions in Southeast Asia.

## Introduction

The domestication of wild animals represents a major milestone in the course of human civilisation^1^. Throughout human history, approximately 40 livestock species have been domesticated, most of which still contribute to agriculture and food production as domestic animals today^2^. The chicken is one of the most ubiquitous domesticated animals; it is bred for both its egg and meat, and is thought to have originally been domesticated from the red junglefowl (*Gallus gallus*) native to multiple regions from Southeast Asia to Southwest China^3–5^. Chicken domestication was previously considered to have occurred in the Indus Valley at around 2000 BC^6^_._ However, West and Zhou^7^ proposed an earlier origin in Southeast Asia, before the 6000 BC, based on archaeological evidence from China, Southeast Asia, and Europe, and palaeoclimatic evidence in China. Chickens were initially used for rituals, including the use of a crowing cock to proclaim the hour of dawn, and later, various cock-fighting and pet breeds were produced and raised around the globe. Commercial chicken breeds, including layers and broilers, have been bred over the last 100 years through selective mating of various indigenous breeds^8–11^.

The process of chicken domestication through the breeding of indigenous chickens has an approximately 8000 year-long history. Consequently, over the years, indigenous chickens have acquired diverse genetic characteristics that have facilitated adaptation to different challenging conditions in diverse locations^12^, such as heat stress, humidity^13–15^, and disease^16^. Recently, the crossbreeding of indigenous chickens with common commercial chickens has been actively performed to develop breeds that exhibit the aforementioned desirable traits. However, repeated crossing with commercial chickens decreases the genetic diversity of indigenous chickens, resulting in the loss of genetic resources in the latter populations^17^. In addition, in Southeast Asia, genetic contamination of the ancestral species via crossing of red junglefowl with free-range indigenous chickens has become a major concern for conservation biologists. Consequently, genetic characterisation of red junglefowl and indigenous chickens is essential to reveal the potential genetic erosion in the former. In addition, the results of such activities could facilitate the adoption of appropriate strategies to recover and conserve the genetic diversity of the species, which represents an invaluable genetic resource.

Population studies on the genetic diversity and relationship between red junglefowl and indigenous chickens could also provide key insights into the geographic origins of chicken domestication and genome evolution in chickens over the course of domestication. Numerous studies on the genetic diversity of chickens have been conducted using mitochondrial DNA (mtDNA) D-loop sequences^4,5,18–23^. Miao et al.^5^ identified five major (A–E), six minor (F–J), and several rare (W–Z) haplogroups based on mtDNA D-loop sequences in 206 red junglefowls from eight countries (China, India, Indonesia, Laos, Myanmar, Philippines, Thailand, and Vietnam) and 3797 indigenous chickens from 30 countries in Asia, Europe, Africa, South America, and six Pacific islands. In Miao et al.^5^ and other reports cited in their study^18,19,23^, 32 red junglefowls (AB009432 and AB009441 in Miyake et al., 1997, direct submission to the international database GenBank [NCBI], HM462194 to HM462217^18^, D82900, D829001, and D82906 to D82908^19^) and indigenous chicken individuals (AB007724 in Miyake et al., direct submission to the international database GenBank [NCBI]; D82922^19^; FJ914360 and FJ914361^23^) from Thailand were included. In addition, Teinlek et al.^24^ recently examined the mtDNA D-loop sequences of 220 Thai indigenous chickens consisting of four breeds (Pradu-hang-dam, Leung-hang-khao Chee, and Dang). Although Thailand may be one of the regions where chicken domestication occurred^3^, the data regarding the genetic diversity of red junglefowls and indigenous chickens in Thailand are still insufficient to accurately elucidate the origin and process of chicken domestication. Red junglefowl mainly inhabit Southeast Asia, and five subspecies (*Gallus gallus spadiceus*, *G. g. gallus*, *G. g. jabouillei*, *G. g. bankiva*, and *G. g. murghi*) have been reported^25,26^. Two subspecies, *G. g. gallus* and *G. g. spadiceus*, distributed across Thailand^25,26^, are distinguished only by their external characteristics, and no population genetics study on the genetic diversity between the two subspecies has been carried out. In Thailand, red junglefowl is strictly protected under the National Park Act since 1961 and the revised Wild Animals Reservation and Protection Act since 1992^27^. Thai indigenous chickens are generally reared under free range backyard conditions by small-scale poultry farmers, and a variety of breeds have been established from indigenous populations and maintained as closed colonies ever since. These were bred selectively based on their morphological and/or biological characteristics, such as meat and/or egg production, quality of meat, plumage or morphologies, behaviors (crowing and fighting), etc., without genetic contamination or gene flow from other breeds or populations. The price of eggs and meat from these indigenous chickens is relatively high compared to those of commercial layers and broilers. Ornamental and fighting chickens are also traded at a higher price among chicken fanciers. Because indigenous chickens are a major or secondary source of income for poultry farmers, collecting blood or skin samples from indigenous chicken breeds and red junglefowl is difficult.

Here, we conducted a large-scale population genetics study on the diversity of red junglefowl (279 individuals from 12 populations) and indigenous chicken populations (138 chickens from 10 indigenous chicken breeds) in Thailand using mtDNA D-loop sequences and microsatellite markers to investigate (1) the mtDNA D-loop haplotypes of red junglefowl and indigenous chickens in Thailand; (2) the existence of ancient D-loop sequence haplotypes in red junglefowl, which would indicate a likelihood that Thailand is one of the centres of chicken domestication; (3) the genetic differentiation between two red junglefowl subspecies, *G. g. gallus* and *G. g. spadiceus*; and (4) the current genetic structure of red junglefowl and indigenous chickens in Thailand. On the basis of our results, we discuss the origin of indigenous chicken breeds in Thailand and the genomic evolution that has occurred in the populations over the course of domestication.

## Results

### Determination of the mtDNA D-loop haplotypes of indigenous chicken breeds and red junglefowl in Thailand

**Figure 1:**
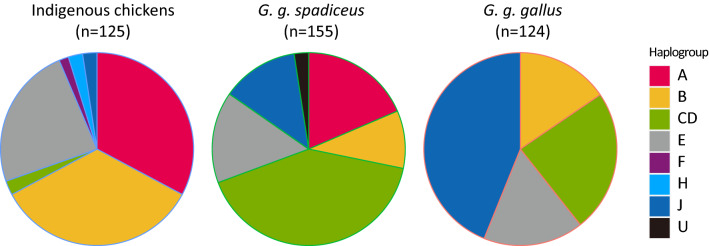
Composition ratio of haplogroups of the mtDNA D-loop sequences in chickens indigenous to Thailand, *G. g. spadiceus*, and *G. g. gallus*. The haplogroup names were conformed to those described by Miao et al.^5^ The numbers in parentheses indicate the number of individuals examined.

We determined the nucleotide sequences of the 780 bp fragments of the mtDNA D-loop region, including the hypervariable segment I, in 125 individuals from 10 indigenous chicken breeds (a list of breeds is shown in Table 1), and 279 red junglefowls from two subspecies (*G. g. gallus* and *G. g. spadiceus*) within 12 populations in Thailand. A total of 44 haplotypes with 62 variable sites, consisting of 26 singletons and 36 parsimony informative sites were identified (Supplementary Tables S1, S2; accession No. LC542982 to LC543385). Table 2 summarizes the details of the haplotypes found in the 10 indigenous chicken breeds and 12 red junglefowl populations, and Fig. 1 shows the composition of each haplogroup of indigenous chickens and two subspecies of red junglefowl. Forty-four haplotypes were temporally classified into eight common haplogroups; A, B, C, D, E, F, H, and J (Supplementary Figs. S1, S2), according to Liu et al.^4^ and Miao et al.^5^. In the present study, we treated haplogroups C and D as one unit (CD) as they were not clearly separated. In addition, haplogroup J was closely related to haplogroup CD. Haplogroups A, B, and E were predominant in the Thai indigenous chickens (Table 2), and their frequencies were almost the same (Fig. 1). Haplogroup CD was predominant in the *G. g. spadiceus* population, but rare in indigenous chickens. In the *G. g. gallus* population, the haplogroups B, CD, and E were detected at almost the same frequency; however, haplogroup A was not detected. The frequency of haplogroup J, which was mainly found in the Si Sa Ket population, was much higher in *G. g. gallus* compared with indigenous chicken and *G. g. spadiceus* populations (Fig. 1). BT, NK-W, NK-B, LHK, CH, PHD, Decoy, fighting chicken, and seven red junglefowl populations (Huai Sai [Ggg], Huai Sai [Ggs], Sa Kaeo, Chanthaburi, Khao Kho, Chaiyaphum, and Khok Mai Rua) each exhibited breed- or population-specific haplotypes (Supplementary Table S2): Hap_04 (BT); Hap_07 and 08 (NK-W); Hap_09 (NK-B); Hap_10 and Hap_11 (LHK); Hap_14 (CH); Hap_17 (PHD); Hap_18 to 20 (Decoy); Hap_29 to 33 (fighting chicken); Hap_21 (Huai Sai [Ggg]); Hap_22 (Huai Sai [Ggs]); Hap_24 and 25 (Sa Kaeo); Hap_27 and 28 (Chanthaburi); Hap_36 (Khao Kho); Hap_38 and 39 (Chaiyaphum); and Hap_43 and 44 (Khok Mai Rua). Twenty-nine out of 44 D-loop haplotypes which had not been previously deposited in GenBank, were newly identified in the present study.

**Table 1:** List of indigenous chicken breeds and red junglefowl populations examined in the present study.

Category	Breed/population	Abbreviation	Location of sampling	No. of individuals examined
Indigenous chicken breed	Lueng-hang-khao	LHK	Phitsanulok	17
	Chee	CH	Phitsanulok	10
	Pradu-hang-dam	PHD	Phitsanulok	10
	Kheaw-Paree	KP	Phitsanulok	10
	Betong	BT	Lopburi	30
	Decoy	Decoy	Phitsanulok, Sukhothai, ChaingMai	6
	Fighting chicken	–	Bangken	30
	Nin Kaset (white)	NK-W	Lopburi	10
	Nin Kaset (black)	NK-B	Lopburi	10
	Dong-Tao	DT	Lopburi	5
Red junglefowl	Sa Kaeo (*G. g. gullus*)	–	Sa Kaeo	30
	Chanthaburi (*G. g. gullus*)	–	Chanthaburi	30
	Si Sa Ket (*G. g. gullus*)	–	Si Sa Ket	30
	Roi Et (*G. g. gullus*)	–	Roi Et	30
	Khok Mai Rua (*G. g. gallus*)	–	Khok Mai Rua	30
	Chaing Rai (*G. g. gallus*)	–	Chaing Rai	9
	Huai Sai (*G. g. gullus*)	Huai Sai (Ggg)	Huai Sai	4
	Huai Sai (*G. g. spadiceus*)	Huai Sai (Ggs)	Huai Sai	15
	Khao Kho (*G. g. spadiceus*)	–	Khao Kho	30
	Chaiyaphum (*G. g. spadiceus*)	–	Chaiyaphum	30
	Petchaburi (*G. g. spadiceus*)	–	Petchaburi	30
	Huai Yang Pan (*G. g. spadiceus*)	–	Huai Yang Pan	30

**Table 2:** Summary of haplogroups of mtDNA D-loop sequences and haplotypes that were found in 10 indigenous chicken breeds and 12 populations of two *Gallus gallus* subspecies in Thailand and their distribution.

Haplogroup	Haplotype	Indigenous chicken breed	Red junglefowl population	
			*G. g. gallus*	*G. g. spadiceus*
A	Hap_03, Hap_12, Hap_18–20, Hap_30	LHK, CH, PHD, KP, BT, Decoy, fighting chicken	–	Huai Sai, Petchaburi
B	Hap_01, Hap_02, Hap_14, Hap_16, Hap_21, Hap_29, Hap_33	LHK, CH, PHD, KP, Decoy, fighting chicken, DT	Sa Kaeo, Huai Sai	Huai Sai, Khao Kho, Petchaburi
CD	Hap_17, Hap_22, Hap_23, Hap_27, Hap_28, Hap_31, Hap_32, Hap_36, Hap_37, Hap_41, Hap_43, Hap_44	PHD, fighting chicken	Chanthaburi, Khok Mai Rua, Chaing Rai	Huai Sai, Khao Kho, Chaiyaphum, Huai Yang Pan
E	Hap_04, Hap_06–09, Hap_15, Hap_35, Hap_39	CH, BT, NK-W, NK-B	Roi Et, Khok Mai Rua	Khao Kho, Chaiyaphum, Huai Yang Pan
F	Hap_13	LHK, PHD	–	–
H	Hap_05	BT, fighting chicken	–	–
J	Hap_10, Hap_11, Hap_24–26, Hap_34, Hap_40, Hap_42	LHK, fighting chicken	Sa Kaeo, Chabthaburi, Si Sa ket, Roi Et, Khok Mai Rua, Chaing Rai	Huai Yang Pan
Unclassified (U)	Hap_38	–	–	Chaiyaphum

The topologies of the Bayesian tree and the maximum-likelihood (ML) tree based on the HKY + G + I model of evolution, which were selected as the best-fit substitution model, were fundamentally similar. Although the Bayesian posterior probability of the internal nodes and the ML bootstrap values were relatively low due to the short internal branches (multifurcations) of the phylogenetic trees, the haplogroups A, B, F–I, K, Y, and Z were supported by a Bayesian posterior probability of greater than 0.97 (Supplementary Figs. S1, S2). Both The trees revealed that the D-loop sequences obtained in this study could be classified into six haplogroups: A, B, CD, E, F, and J, and a complex group of rare haplogroups (H, I, K, W, and X), except for an unclassified haplotype, Hap_38.

**Figure 2:**
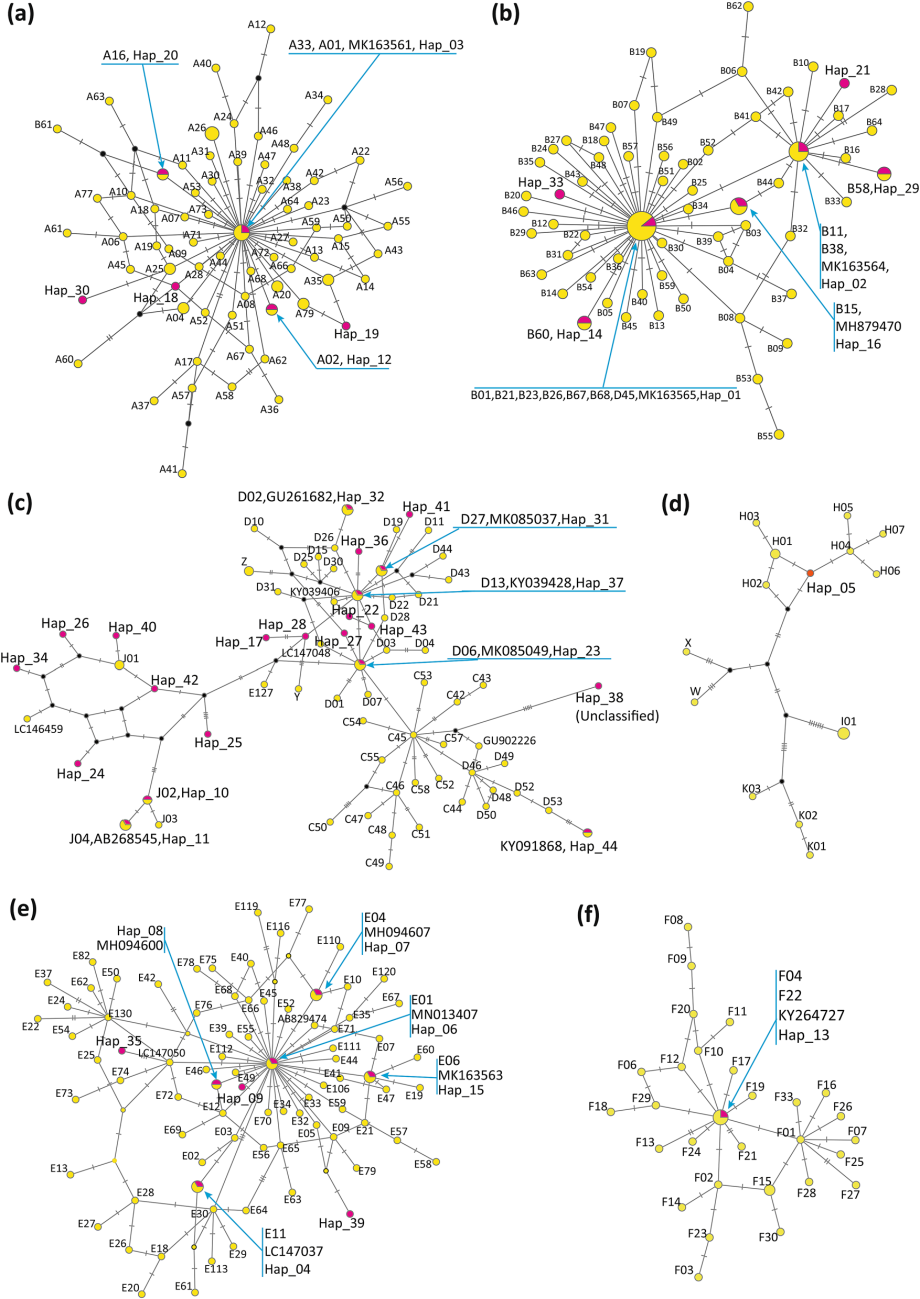
Locations of mtDNA D-loop haplotypes of Thai red junglefowl and indigenous chicken populations in the global chicken population network. (**a**) Haplogroup A. (**b**) Haplogroup B. (**c**) Haplogroups CD, Y, Z, J, and an unclassified haplotype, Hap_38. (**d**) Haplogroups H, I, K, X, and W. (**e**) Haplogroup E. (**f**) Haplogroup F. Haplotypes that were found in the present study and representative haplotypes reported by Miao et al*.*^5^ are shown by magenta and yellow circles, respectively. Black nodes are the inferred intermediate haplotypes. The number of bars on the lines, which link haplotypes, represent the number of nucleotide substitutions that occurred between the haplotypes for comparison.

Six haplotypes from seven indigenous and two red junglefowl populations (LHK, CH, PHD, KP, BT, Decoy, fighting chicken, Huai Sai [Ggs], and Petchaburi) belonged to haplogroup A (Fig. 2a). Seven haplotypes from seven indigenous chicken breeds and five red junglefowl populations (LHK, CH, PHD, KP, Decoy, fighting chicken, DT, Sa Kaeo, Huai Sai [Ggg], Huai Sai [Ggs], Khao Kho, and Petchaburi) were classified into haplogroup B (Fig. 2b). Haplogroup CD contained 12 haplotypes, which were identified in two Thai indigenous chicken breeds (PHD and fighting chicken) and seven red junglefowl populations (Chanthaburi, Khok Mai Rua, Chaing Rai, Huai Sai [Ggs], Khao Kho, Chaiyaphum, and Huai Yang Pan) (Fig. 2c). Eight haplotypes in four indigenous chicken breeds (CH, BT, NK-W, and NK-B) and four red junglefowl populations (Roi Et, Khok Mai Rua, Chaiyaphum, and Huai Yang Pan) belonged to haplogroup E (Fig. 2e). Haplogroup F contained one haplotype, which was only found in two indigenous chicken breeds (LHK and PHD) (Fig. 2f). Eight haplotypes of haplogroup J (Hap_10, Hap_11, Hap_24 to 26, Hap_34, Hap_40, and Hap_42) were found in two indigenous chicken breeds (LHK and fighting chicken) and seven red junglefowl populations (Sa Kaeo, Chabthaburi, Si Saket, Roi Et, Khok Mai Rua, Chaing Rai, and Huai Yang Pan) (Fig. 2c). Only one haplotype of haplogroup H (Hap_05) was detected in two indigenous chicken breeds (BT and fighting chicken) (Fig. 2d). Hap_38, which was found in three individuals of the Chaiyaphum population, did not belong to any known haplogroups; however, the haplotype was more closely related to haplogroup CD than the other haplogroups (Fig. 2c; Supplementary Figs. S1, S2).

Divergence times for each haplotype were determined using BEAST analysis (Supplementary Table S2) and were 0.24–0.45 kilo years ago (KYA) for haplogroup A, 0.15–0.39 KYA for haplogroup B, and 0.14–0.37 KYA for haplogroup CD; the haplotypes of haplogroup E exhibited a wide range of divergent times, ranging from 0.12 to 0.70 KYA (0.41 KYA on average). One haplotype in haplogroups F and H had possibly diverged at 0.33 and 0.34 KYA, respectively. The divergence times of haplotypes in haplogroup J ranged from 0.10 to 0.60 KYA. Hap_38 exhibited a markedly earlier divergence time, which was estimated to be approximately 12,000 years ago (Supplementary Table S2; Supplementary Fig. S1).

### Genetic diversity of mtDNA D-loop sequences

The number of D-loop haplotypes in each population (*H*) ranged from 1 (*G. g. gallus* population at Huai Sai and Si Sa Ket) to 10 (fighting chicken) (Table 3). Among the Thai indigenous chicken breeds, LHK, CH, PHD, KP, BT, Decoy and fighting chicken exhibited relatively higher genetic diversity (*pi*, 0.005 for KP and Decoy to 0.009 for LHK, PHD, and fighting chicken; *Theta-w*, 2.86 for KP to 8.30 for fighting chickens) than in NK-W, NK-B, and DT (*pi*, 0.001 for NK-W, NK-B, and DT; *Theta-w*, 0.35 for NK-B to 0.71 for NK-W) (Table 3). With regard to the red junglefowl populations, all populations excluding Si Sa Ket and Petchaburi exhibited similar levels of genetic diversity. The Petchaburi population had two haplotypes, and the genetic diversity was relatively low. The low genetic diversity in the Si Sa Ket population was attributed to the fact that all 30 examined individuals shared only one haplotype. Seven out of the 10 indigenous chicken breeds (LHK, CH, PHD, Decoy, fighting chicken, NK-W, and DT) exhibited negative *Tajima’s D* values, suggesting that the chickens were bred under purifying selection within each population; however, even though the *Tajima’s D* values of all populations were not statistically significant (p > 0.05).

**Table 3:** Genetic diversity of indigenous chicken breeds and red junglefowl populations estimated using of mtDNA D-loop sequences and 28 microsatellite markers.

Category	Location	mtDNA D-loop sequence					Microsatellite marker						
		*N*	*H*	*pi*	*Theta-w*	Tajima's D^a^	*N*	*AR*	*MNA*	*Ne*	*He*	*Ho*	*F*
Indigenous chicken breed	Lueng-hang-khawo (LHK)	15	6	0.009	7.07	− 0.54	17	3.25	5.18	3.06	0.62	0.57	0.07
	Chee (CH)	9	5	0.007	5.52	− 0.26	10	3.17	4.21	2.98	0.61	0.56	0.06
	Pradu-hang-dam (PHD)	9	5	0.009	6.99	− 0.39	10	2.89	3.82	2.54	0.57	0.51	0.12
	Kheaw-Paree (KP)	7	2	0.005	2.86	1.98	10	3.17	4.46	2.84	0.59	0.59	0.02
	Betong (BT)	30	3	0.008	3.79	1.15	30	2.26	3.32	2.01	0.43	0.45	− 0.05
	Decoy	6	5	0.005	4.38	− 1.12	6	3.24	3.68	2.85	0.59	0.52	0.10
	Fighting chicken	24	10	0.009	8.30	− 0.67	30	3.19	6.07	3.25	0.60	0.60	0.01
	Nin Kaset (white) (NK-W)	10	2	0.001	0.71	− 0.69	10	2.83	3.54	2.50	0.56	0.60	− 0.07
	Nin Kaset (black) (NK-B)	10	2	0.001	0.35	0.82	10	2.76	3.43	2.50	0.55	0.64	− 0.16
	Dong-Tao (DT)	5	2	0.001	0.48	− 0.82	5	2.96	3.43	2.52	0.53	0.54	− 0.02
Red junglefowl	Sa Kaeo (*G. g. gallus*)	30	3	0.006	3.79	0.66	30	3.14	4.93	3.23	0.61	0.59	0.04
	Chanthaburi (*G. g. gallus*)	30	3	0.006	2.27	2.37	30	3.59	6.29	3.94	0.67	0.68	0.01
	Si Sa Ket (*G. g. gallus*)	30	1	0.000	0.00	0.00	30	3.05	5.39	2.98	0.59	0.58	0.03
	Roi Et (*G. g. gallus*)	30	2	0.007	3.28	1.54	30	2.78	4.29	2.63	0.53	0.51	0.06
	Khok Mai Rua (*G. g. gallus*)	27	6	0.007	4.41	0.59	30	3.48	7.07	3.49	0.66	0.60	0.11
	Chaing Rai (*G. g. gallus*)	6	3	0.006	3.50	1.96	9	3.34	4.21	3.21	0.63	0.66	− 0.04
	Huai Sai (*G. g. gallus*)	2	1	0.000	0.00	n/a	4	2.81	2.82	2.37	0.51	0.45	0.10
	Huai Sai (*G. g. spadiceus*)	13	4	0.008	4.83	0.72	15	3.32	4.96	3.21	0.65	0.56	0.13
	Khao Kho (*G. g. spadiceus*)	30	4	0.007	3.79	0.60	30	3.33	5.29	3.31	0.66	0.64	0.05
	Chaiyaphum (*G. g. spadiceus*)	30	4	0.002	2.02	− 0.72	30	3.39	5.29	3.55	0.66	0.66	0.02
	Petchaburi (*G. g. spadiceus*)	23	2	0.004	1.90	1.54	30	3.17	5.00	3.01	0.63	0.64	− 0.01
	Huai Yang Pan (*G. g. spadiceus*)	28	4	0.006	3.60	0.82	30	2.99	5.04	2.84	0.58	0.57	0.01
	Total	404					436						

*N*: Number of individuals examined. *H*: Number of observed haplotypes. *pi*: Nucleotide diversity. *Theta-w*: Watterson estimator (*Theta-w* per sequence in Dnasp). *AR*: Allelic richness, *MNA*: Mean number of allesles per locus, *Ne*: Number of effective alleles frequencies = 1/(Sum *pi*^2^). *He*: Expected heterozygosity. *Ho*: Observed heterozygosity. *F*: Fixation index = (*He* − *Ho*)/*He.*

^a^No statistical significance was detected for all of 22 populations.

### Phylogenetic relationships among mtDNA D-loop haplotypes in red junglefowl in Asia

**Figure 3:**
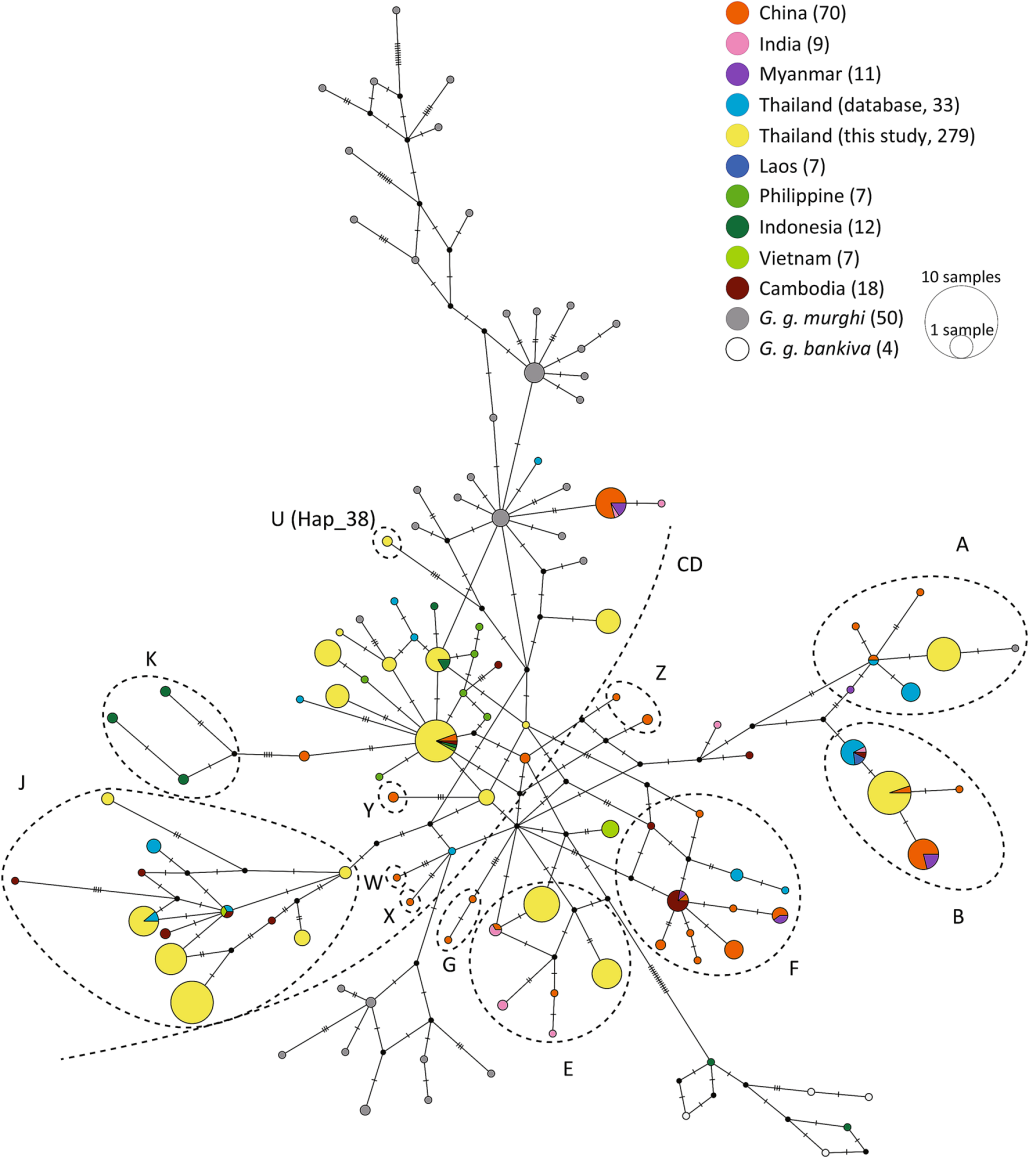
Median-joining haplotype network of mtDNA D-loop sequences of red junglefowl. The haplotypes are approximately subdivided into 12 haplogroups, A, B, CD, E, F, G, J, K, W, X, Y, and Z, and unclassified haplotypes (U) in this network, according to the haplotype classification by Miao et al.^5^ and the present study. The sizes of circles indicate relative frequencies of haplotypes, and the number of bars on the lines, which link haplotypes, represent the number of nucleotide substitutions that occurred between the haplotypes for comparison. Black nodes are the inferred intermediate haplotypes. The geographic origins of haplotypes or subspecies names are shown using circles with different colours. The numbers in parentheses after the location or subspecies names indicate the numbers of sequences used for analyses. Detailed information on the sequences obtained from the database are listed in Supplementary Table S7.

Haplogroups A, B, CD, E, and J were frequently identified in red junglefowl in Thailand (Fig. 1; Supplementary Table S2). Haplotypes from Thailand, Vietnam, Laos, and Myanmar were located in internal nodes of the haplogroup A, B, CD, and E, and haplotypes from China were derived from the haplotypes in Southeast Asia (Fig. 3). Haplogroup J exclusively consisted of haplotypes from Thailand, Vietnam, and Cambodia. In haplogroup F, haplotypes from Cambodia exhibited the ancestral haplotypes of Chinese red junglefowl. Three haplotypes of red junglefowl from Indonesia were observed in haplogroup K. A small number of haplotypes from the other rare haplogroups G and W to Z were only observed in Chinese red junglefowl.

### Genetic characteristics of indigenous chicken breeds and red junglefowl estimated by 28 microsatellite DNA markers

Two-hundred and ninety-eight red junglefowls in two subspecies (*G. g. gallus* and *G. g. spadiceus*) from 12 populations and 138 chickens from 10 indigenous chicken breeds, were used for the genetic diversity analyses using 28 microsatellite markers (Table 1; Supplementary Table S3). The allelic richness (*AR*) values ranged from 1.40 for MCW103 to 1.93 for MCW0014 (1.77 on average) (Supplementary Table S3). *Na* ranged from 2.14 for MCW0103 to 7.59 for LEI0192 (2.86 on average). *F*_*IS*_ varied from – 0.08 for LEI0166 to 0.54 for MCW0014 (0.05 on average). The *F*_*ST*_ and *F*_*IT*_ values fell within the 0.07 (MCW0098) to 0.31 (MCW0247) range and 0.09 (MCW0123) to 0.63 (MCW0014) range, respectively (*F*_*ST*_ = 0.17, *F*_*IT*_ = 0.22 on average). Two markers, MCW0222 and MCW0014, showed the null allele frequency across all populations (*NAF*) higher than 0.2 (Supplementary Table S3). Looking at each population, null allele frequencies higher than 0.2 were detected in seven, three, and three populations for MCW0014, MCW0222, and LEI0192, respectively, and in less than one or two populations for the other 11 markers (Supplementary Table S4).

Significant departures from Hardy–Weinberg equilibrium were observed for LEI0234, MCW0014, and MCW0123 in more than 10 populations (p < 0.05) (Supplementary Table S5). The Khok Mai Rua and Huai Sai (Ggs) populations exhibited significant departures at 13 and 11 loci, respectively, while the other red junglefowl and indigenous chicken populations exhibited significant departures at less than 10 loci (p < 0.05).

Out of the 22 populations examined, the BT population exhibited the least genetic diversity (*AR* = 2.26; mean number of alleles [*MNA*]) 3.32; *Ne* = 2.01; *Ho* = 0.45) (Table 3). In the other nine indigenous chicken breeds, *AR* ranged from 2.76 (NK-B) to 3.25 (LHK); MNA ranged from 3.43 (NK-B and DT) to 6.07 (fighting chicken); *Ne* ranged from 2.50 (NK-W and NK-B) to 3.25 (fighting chicken); and *Ho* ranged from 0.51 (PHD) to 0.64 (NK-B). In the red junglefowl populations, *AR* ranged from 2.78 (Roi Et) to 3.59 (Chanthaburi); *MNA* ranged from 2.82 (Huai Sai [Ggg]) to 7.07 (Khok Mai Rua); *Ne* ranged from 2.37 (Huai Sai [Ggg]) to 3.94 (Chanthaburi); and *Ho* ranged from 0.45 (Huai Sai [Ggg]) to 0.68 (Chanthaburi). Chanthaburi (*AR* = 3.59; *MNA* = 6.29; *Ne* = 3.94; *Ho* = 0.68) and Khok Mai Rua (*AR* = 3.48; *MNA* = 7.07; *Ne* = 3.49; *Ho* = 0.60) exhibited comparatively higher genetic heterogeneity among the populations examined.

### Genetic relationships among indigenous chicken breeds and red junglefowl in Thailand

**Figure 4:**
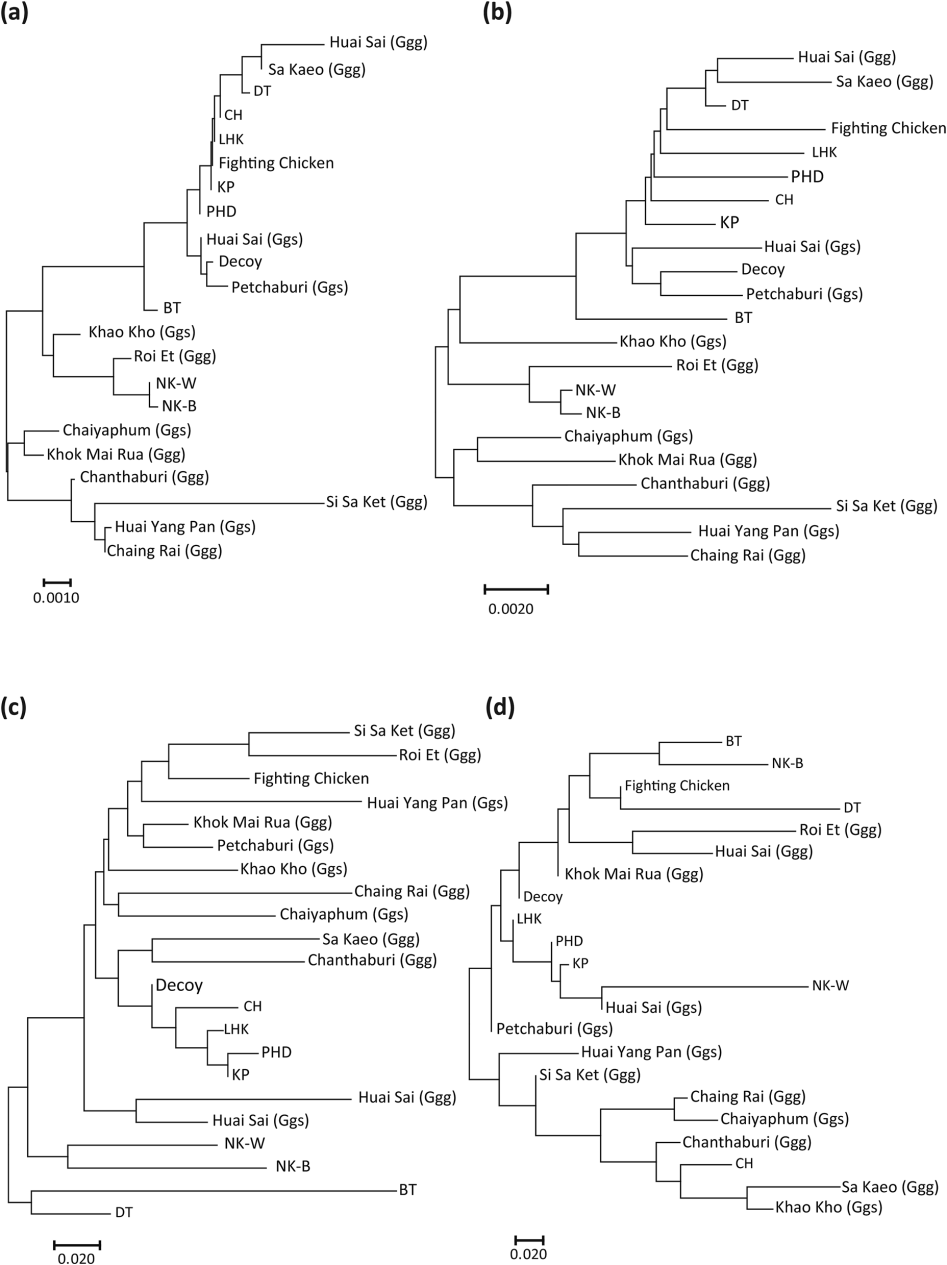
Neighbour-joining trees of 10 indigenous chicken breeds and 12 red junglefowl populations in Thailand constructed using mtDNA D-loop sequences and microsatellite markers. Neighbour-joining trees were constructed using Nei’s genetic distance according to allele frequencies (*Da*) (**a**) and the genetic distance based on nucleotide substitution per site (*Dxy*) (**b**) using mtDNA D-loop sequences and according to pairwise *F*_*ST*_ distance (**c**) and pairwise *R*_*ST*_ distance (**d**) using 28 microsatellite markers.

Two cladograms based on *Da* and *Dxy* genetic distances constructed with mtDNA D-loop sequences exhibited similar topologies (Fig. 4a,b); six breeds of Thai indigenous chickens (LHK, CH, PHD, KP, Decoy, and fighting chicken) and the DT chicken breed showed close genetic relationships in both trees, whereas three other Thai indigenous chicken breeds (BT, NK-W and NK-B) were phylogenetically located far from the six breeds. Two red junglefowl subspecies were not phylogenetically separated from each other. However, the phylogenetic tree based on the *F*_*ST*_ genetic distance constructed using microsatellite markers indicated that all red junglefowl populations excluding Huai Sai (Ggg) and Huai Sai (Ggs) had a close genetic relationship (Fig. 4c). Five indigenous chicken breeds (Decoy, CH, LHK, PHD, and KP) fell in a cluster distinct from the red junglefowl populations; however, the fighting chickens were genetically closer to red junglefowl. Four indigenous chicken breeds (BT, NK-W, NK-B and DT) were obviously genetically different from the other indigenous chicken breeds and the red junglefowl populations. The phylogenetic tree topology based on *R*_*ST*_ genetic distances was largely different from those based on *Da*, *Dps*, and *F*_*ST*_ genetic distances (Fig. 4d). In the unrooted phylogenetic trees among individuals based on *Da* and *Dps* genetic distances, most of the 22 populations were classified into distinct clusters (only the tree based on the *Da* genetic distance is presented in Supplementary Fig. S3). While BT, NK-W, NK-B, and DT each formed a distinct cluster, the LHK, CH, PHD, and KP individuals formed a mixed cluster, where a few Decoy and DT individuals were included. The clusters of all the red junglefowl populations were basically distinct, although no obvious genetic differentiation was observed between the two subspecies, *G. g. gallus* and *G. g. spadiceus* (illustrated by open and filled circles, respectively).

**Figure 5:**
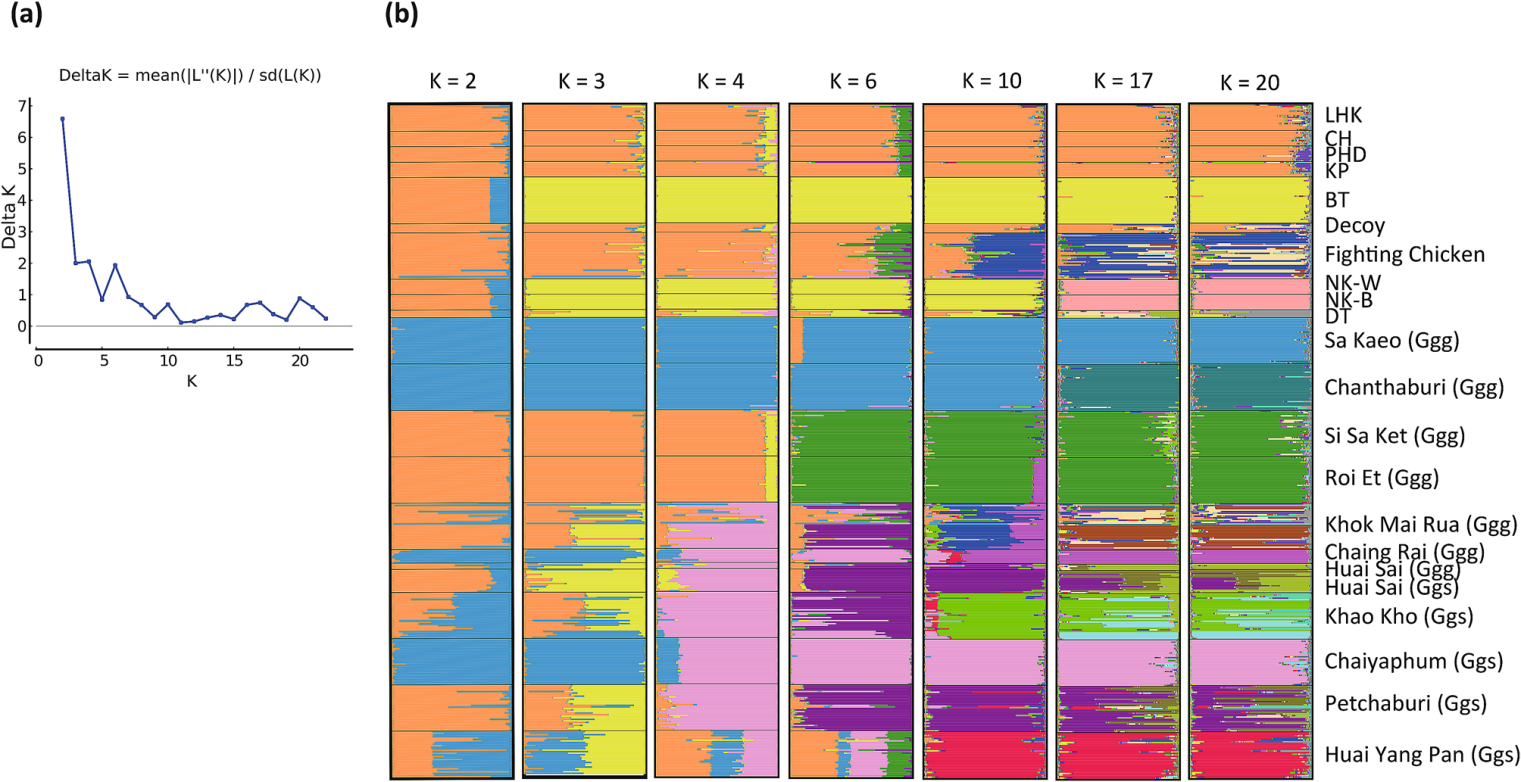
Bayesian clustering of 10 indigenous chicken breeds and 12 red junglefowl populations in Thailand. (**a**) Delta K values at K = 2 to K = 23. (**b**) Group memberships of 10 indigenous chicken breeds and 12 red junglefowl populations at K = 2, 3, 4, 6, 10, 17, and 20 are shown in different colours.

We performed STRUCTURE analysis using 26 MS markers, excluding two markers, MCW0222 and MCW0014, which showed *NAF* higher than 0.2; however, we realized that the resulting STRUCTURE plots did not differ from those obtained using all 28 markers. Thus, we used all of the 28 markers for STRUCTURE analysis in this study. Analysis using STRUCTURE HARVESTER revealed that K = 2 was optimal for the 22 populations (delta K = 6.59) and K = 4 was the second highest (delta K = 2.05) (Fig. 5a). The STRUCTURE plot at K = 2 subdivided four red junglefowl populations (Sa Kaeo, Chanthaburi, Chaing Rai, and Chaiyaphum) from the other red junglefowl populations and indigenous chicken breeds. The 22 populations were classified into four clusters at K = 4 as follows: (1) indigenous chickens, such as ornamental chicken breeds (LHK, CH, PHD, and KP), Decoy, fighting chicken, and two red junglefowl populations (Si Sa Ket and Roi Et); (2) indigenous meat chicken breeds (BT and two NK breeds) and DT; (3) two red junglefowl populations (Sa Kaeo and Chanthaburi); and (4) the other populations of red junglefowl, consisting of Khok Mai Rua, Chaing Rai, Huai Sai (Ggg), Huai Sai (Ggs), Khao Kho, Chaiyaphum, Petchaburi, and Huai Yan Pan (Fig. 5b). However, Huai Yang Pan and Khok Mai Rua were mixed with other populations, such as Si Sa Ket and Roi Et, as well as Sa Kaeo and Chanthaburi. At K = 6, two populations of red junglefowl (Si Sa Ket and Roi Et) formed a cluster independent from the other six Thai indigenous chicken breeds (LHK, CH, PHD, KP, Decoy, and fighting chicken).

Analysis of molecular variance (AMOVA) revealed that 83.85% and 16.00% of the total genetic variance in 12 red junglefowl populations was attributed to genetic variance within populations and among groups of each subspecies, respectively, and only 0.16% of the total genetic variance was attributed to variance between two subspecies. This result indicates that the genetic variation of red junglefowl is mostly explained by the genetic divergence among populations, and that the genetic difference between subspecies is very low.

## Discussion

Haplogroups A, B, and CD of mtDNA D-loop sequences are mainly distributed in chickens from East Asia, Southeast Asia, and the Pacific islands^4,5,28^. The haplotypes of haplogroups A and/or CD that were observed in 15 populations of indigenous chickens and red junglefowl in Thailand were similar or closely related to the representative haplotypes in South China, Vietnam, Laos, Sri Lanka, and Japan^5^. Some haplotypes of haplogroups F and J, which are minor haplogroups found only in limited areas of Southern China and Southeast Asia^4,5,21,22,29^, were also found in Thai indigenous chickens. However, 29 unknown haplotypes were newly identified in the present study, with 14 and 17 identified in indigenous chickens and red junglefowl, respectively. Indigenous chickens and red junglefowl shared two haplotypes (Hap_12 and 34). Most of their divergence times were estimated to be around 300 years ago or earlier. Our results indicate that the unique genetic variants have been maintained in the indigenous chicken populations for a long time after the original domestication of red junglefowl in the region.

Notably, the identification of haplogroup H, which was found in fighting chickens, is a key piece of evidence supporting the historical link between Thailand and Japan through cock-fighting. Cock-fighting is a traditional game that is historically widespread across the world, but especially common in Southeast Asia; the breed specialised for the game is called ‘Shamo’ in Japan. Currently, this breed is popular as a fancy breed. In a previous study on the genetic characteristics of Japanese fighting chickens using mtDNA D-loop sequences^30^, the Japanese Shamo chicken was clearly separated into two phylogenetic groups: one group from the Okinawa islands and the other group mainly from Kyushu and Honshu. This result suggests that the Japanese Shamo chicken has two origins. Trade between Thailand and Japan, especially the Okinawa islands, was active during the Ayutthaya Kingdom (circa the 15th–16th century)^30^, and the name ‘Shamo’ in Japanese is proposed to have been derived from ‘Siam’ or ‘Sa-yam’, which was the old name of Thailand used in this era. Our results strongly support the view that Thailand is one of the origins of the Japanese Shamo chicken.

The genetic diversity of Thai indigenous chicken breeds and their phylogenetic relationships, as examined using microsatellite markers, revealed that the chicken breeds bred for meat were genetically distinct from the other indigenous chicken breeds. The BT, NK-B, NK-W, and DT chicken breeds were each divided into a separate cluster. The results suggest that these breeds have a long history of selective breeding, which has led to the emergence of unique genetic characteristics in all four breeds. As shown in the STRUCTURE plot, four indigenous ornamental chicken breeds (LHK, CH, PHD, and KP) and the Decoy chicken were clustered into the same group possibly because (1) the four breeds were established by crossbreeding indigenous chicken breeds of different origins which were not genetically distant from each other or (2) they were derived from a common single breed and consequently had not yet clearly differentiated. The Decoy chicken breed is used for catching wild red junglefowls that are attracted to the crow of Decoy chickens. Decoy chickens have been maintained through inbreeding between Decoy chickens or by mating between Decoy chickens and wild red junglefowl. Therefore, their external appearance is highly varied, and some chickens are similar in appearance to red junglefowl, while others have white or black feathers across their body. This breeding background of Decoy chickens results in a high morphological diversity, but a close genetic relationship between Decoy chickens and red junglefowl populations on the mtDNA level.

The major finding of our study was that six novel J haplotypes (Hap_24–26, Hap_34, Hap_40, and Hap_42) were found in red junglefowl in Thailand; these sequences were not identical to any sequences deposited in GenBank. In addition, a unique haplotype (Hap_38), which has not been classified into any haplogroup, was detected in one wild population (Chaiyaphum), and we postulate that this haplotype diverged from other haplotypes around 12,000 years ago. The results suggest that a part of the wild red junglefowl population gene pool was not involved in the process of domestication. Haplogroups A‒E and J were also the prevailing haplogroups in Thai red junglefowl. Other rare haplogroups, which have been found in many regions in Asia (G, China, Sri Lanka, Laos, and Vietnam; I, India; K, Indonesia; and W–Z, China)^5^, were not found in either the Thai red junglefowl or the indigenous chickens examined in the present study, which suggests that the rare haplogroups are either specific to other regions in Asia, or were not included in the individuals examined in the present study simply by chance. The former would imply that chicken domestication occurred independently in multiple regions of Southeast Asia. Further data on the genetic diversity of red junglefowls from other regions would be required to facilitate a detailed discussion on the origin of indigenous chickens and the process of chicken domestication in Thailand and other Asian countries (Southeast Asia, India, and South China).

According to the STRUCTURE plot analysis, red junglefowl populations were divided into three groups at K = 4, consisting of (1) Sa Kaeo and Chanthaburi; (2) Si Sa Ket and Roi Et; and (3) other populations, excluding Huai Yang Pan. The Si Sa Ket and Roi Et group formed an independent and distinct cluster at K = 6 and 10, and the Khok Mai Rua, Khao Kho, Chaiyapum, and Huai Yang Pan groups each formed independent clusters at K = 10. Si Sa Ket and Roi Et, which were placed in the same cluster at K = 4, are geographically adjacent to each other, as well as to Sa Kaeo and Chanthaburi. However, multiple clusters were found within the Khok Mai Rua population at K = 3 to K = 20. In the phylogenetic tree constructed with microsatellite markers, several individuals of the Khok Mai Rua population were included in other red junglefowl populations. Red junglefowl cannot fly and move over a home range of only approximately 5 ha^31^; however, genetic characteristics were shared between Khok Mai Rua and other populations. This result was consistent with the fact that four of the six D-loop haplotypes of Khok Mai Rua were shared with other population. This suggests that Khok Mai Rua individuals were exchanged between wildlife conservation centres across different provinces. However, there are no records on cross breeding activities between the individuals collected from different populations. Therefore, population-level assessments of the genetic diversity of red junglefowl are required to facilitate the maintenance and preservation of their genetic diversity, in addition to their characteristics in different conservation centres or provinces.

Fumihito et al.^3,19^ proposed a single origin of chicken domestication in and around Thailand inhabited by a subspecies (*G. g. gallus*); however, the theory of multiple origins in South and Southeast Asia was put forward by two comprehensive studies of D-loop sequences by Liu et al.^4^ and Miao et al.^5^ More than 9000 D-loop sequences of chickens have been deposited in GenBank; nevertheless, many novel D-loop haplotypes were discovered from both red junglefowl and indigenous chickens in Thailand in the present study. This result suggests that red junglefowl and indigenous chickens in Thailand have large gene pools with extensive genetic diversity, which has been conserved in the populations for a long time, and that some subset of the red junglefowl population was involved in domestication. Microsatellite markers also exhibited high genetic diversity and unique genetic characteristics of indigenous chickens in Thailand. A recent population genetics study of red junglefowl performed using genome-wide SNP analysis revealed that *G. g. spadiceus* and *G. g. gallus* were phylogenetically differentiated and *G. g. spadiceus* may be the original ancestor of domestic chickens^32^. However, in the present study, the two subspecies were not clearly separated, and the genetic relationships between *G. g. spadiceus* and Thai indigenous chickens were not closer than those between *G. g. gallus* and indigenous chickens. The inconsistency in the results between our study and the previous study could be attributed to the differences in sampling locations of the two subspecies. Although the *G. g. spadiceus* individuals used in the previous study were collected from China, India, and Thailand, six *G. g. gallus* samples were all collected from Indonesia^32^. Given that the two subspecies used in the present study are closely distributed in Thailand, gene flow between different subspecies may readily occur. The small genetic differences between two subspecies in our study suggest that gene flow may have occurred between the populations of the two subspecies.

The results of our study also suggest the possibility that indigenous chickens and red junglefowl in Thailand harbour a variety of diverse genes that regulate traits beneficial to the poultry industry, such as those which improve egg and/or meat production and quality, environmental stress tolerance, and disease resistance. The two subspecies, *G. g. gallus* and *G. g. spadiceus*, are generally distinguished based on earlobe colour^25,26^; however, our results of mtDNA D-loop sequences suggest that there are no major genetic differences between the subspecies in Thailand. The results obtained using mitochondrial DNA, microsatellite DNA, and SNPs on nuclear genomes are affected by population histories due to their differences in the mode of inheritance and mutation rates; therefore, the estimation values of genetic diversity are not necessarily positively correlated between the mtDNA D-loop sequences and microsatellite markers. In comparison to these markers, whole mitochondrial genome sequences will provide a more reliable phylogenetic tree than the D-loop sequences alone. In fact, Miao et al.^5^ mentioned that D-loop sequencing alone could not identify differences between haplogroups C and D and could not subdivide haplogroup E into sub-haplogroups E1–E3. Thus, further genome-wide genetic analysis for red junglefowl and indigenous chickens in Thailand could aid in elucidating the origins and genomic evolution of red junglefowl and domestic chickens, which, in turn, would contribute to the conservation of these invaluable genetic resources.

## Methods

### Ethics statement

The study was conducted under the authority of the Department of National Parks (DNP), Wildlife and Plant Conservation and the Ministry of Natural Resources and Environment, Thailand. Animal care and all experimental procedures were approved by the Animal Experiment Committee at DNP, following the annual physical examination protocol and the Animal Experiment Committee in Kasetsart University (ACKU63-SCI-008). Experiments were conducted in line with the Regulations on Animal Experiments of Kasetsart University and the fundamental guidelines for proper execution of animal experiments and related activities in academic research institutions under the jurisdiction of the National Research Council of Thailand, Ministry of Higher Education, Science, Research and Innovation, Thailand.

### Sample collection and genomic DNA extraction

Two-hundred and ninety-eight individuals of two red junglefowl subspecies from 12 populations were collected from 11 wildlife conservation centres in Thailand (163 individuals of *G. g. gallus* from seven centres and 135 of *G. g. spadiceus* from five centres; one centre provided samples of both subspecies) (Table 1; Supplementary Fig. S4). The subspecies were discriminated based on differences in their morphological traits: *G. g. gallus* have red earlobes, whereas *G. g. spadiceus* have white earlobes^25,26^. Blood samples were collected from 19 individuals in Huai Sai (four *G. g. gallus* individuals and 15 *G. g. spadiceus* individuals), and feather samples were collected from 279 individuals at 11 centres (159 *G. g. gallus* and 120 *G. g. spadiceus* individuals). The 10 indigenous chicken breeds used in the present study include three Thai indigenous chicken breeds for meat production; Betong (BT), Nin Kaset meat chicken with white feathers (NK-W) or black feathers (NK-B), and one indigenous Vietnamese chicken breed for meat production, Dong-Tao (DT), all of which had been maintained at the Lopburi Research Station of Kasetsart University; four fancy chicken breeds, Lueng-hang-khao (LHK), Chee (CH), Pradu-hang-dam (PHD) and Kheaw-Paree (KP) which are bred for poultry show and are identified by their differences in plumage colours; a breed called ‘Decoy’, which was provided by farmers in Phitsanulok province, which are used to catch wild chickens by attracting them by crowing; and fighting chickens, which were provided by a farmer (Table 1). Here, we defined these chickens as indigenous chicken “breeds” for convenience. Blood samples were collected from all the indigenous chickens. Approximately 0.5–1.0 ml blood was collected from the wing veins of live birds using heparinised syringes, and stored in vials containing 5 mM EDTA at 4 °C until use. Genomic DNA was extracted from the basal tips of feather rachises (approximately 1 or 2 mm long) or 20 μl whole blood using commercial DNA extraction kits, ISOSPIN Blood & Plasma DNA (NIPPON GENE, Tokyo, Japan).

### Sequencing of the mtDNA D-loop region and genotyping of microsatellite markers

Partial DNA fragments (780 bp) of the mtDNA D-loop region were amplified by PCR using the following primer set: Gg_Dloop_1F (5′-AGGACTACGGCTTGAAAAGC-3′)^33^, and Gg_Dloop_4R (5′-CGCAACGCAGGTGTAGTC-3′)^34^. Amplification was performed in a 12 μl reaction mixture containing 50 ng genomic DNA, 10 pmol of each primer, and 6.0 μl of AmpliTaq Gold 360 Master Mix (Life Technologies, Tokyo, Japan). The cycling conditions were as follows: initial denaturation at 95 ℃ for 30 min, followed by 35 cycles at 95 ℃ for 30 s, 58 ℃ for 30 s, and 72 ℃ for 30 s, and a final extension for 5 min at 72 ℃. The PCR products were detected by electrophoresis on 1.5% agarose gels and then purified using the 20% polyethylene glycol/2.5 M NaCl precipitation method^35,36^. The cycle sequencing reaction was performed using a Big Dye Terminator Cycle Sequencing Kit v3.1 (Applied Biosystems, Foster City, CA, USA), and nucleotide sequences were determined using an ABI PRISM 3130 Genetic Analyzer (Applied Biosystems). As a preliminary experiment for verifying the reliability of nucleotide sequences of novel or rare haplotypes, we tested the rate of sequencing errors using the same DNA polymerase, instruments and sequencing protocol as those used in this study. We performed 50 independent PCR amplification for the mtDNA D-loop region of one inbred laboratory chicken line (GSP), which was maintained in Avian Bioscience Research Center at Nagoya University. Within the resulted 730 bp D-loop sequences of 50 samples, no sequencing errors were found. Because it is said that the error rate of AmpliTaq Gold 360 polymerase is 10^–5^, sequencing errors of the D-loop region can be almost avoided in this study.

All 28 microsatellite markers used were selected from the 30 markers recommended for chicken biodiversity studies by the Food and Agriculture Organisation^37^ (Supplementary Table S3). PCR amplification was performed using a 12 μl reaction mixture containing approximately 50 ng genomic DNA, 10 pmol of each primer, and 6.0 μl AmpliTaq Gold 360 Master Mix (Life Technologies). The cycling conditions were as follows: initial denaturation at 95 ℃ for 10 min, followed by 35 cycles at 95 ℃ for 30 s, 55 ℃ for 30 s, and 72 ℃ for 30 s, and a final extension for 5 min at 72 ℃. PCR products were electrophoresed with Hi-Di formamide (Applied Biosystems) and GeneScan 600 LIZ Size Standard (Applied Biosystems) using the ABI PRISM 3130 Genetic Analyzer (Applied Biosystems). Allele sizes were determined using GENEIOUS PRIME v2019.2.1 (Biomatters, Auckland, New Zealand).

### Phylogenetic analysis of mtDNA D-loop sequences

DNA sequences were aligned using the Geneious Alignment method implemented in GENEIOUS PRIME v2019.2.1 (Biomatters). Ambiguous sites behind primer sequences were trimmed from the fragments. To determine the phylogenetic positions of red junglefowl and indigenous chickens from Thailand in chicken populations throughout the world, Bayesian and ML phylogenetic trees were constructed using BEAST v2.4.3^38^ and PhyML v3.0^39^ respectively, including 420 D-loop sequences (A01, A02, …Y and Z) obtained from GenBank, which were defined by Miao et al.^5^ (Supplementary Table S6). In addition, 22 D-loop sequences obtained from GenBank were used for analysis (KY039406 and KY039428 in Herrera et al., 2016, direct submission to the international database GenBank; MN013407 in Peng et al., 2019, direct submission to the international database GenBank; other sequences are cited in Supplementary Table S6)^21,22,40–48^. The best-fit substitution model of the D-loop sequences was determined based on the Bayesian Information Criterion using jModeltest v2.1.10^49,50^. Bootstrap support values for ML analyses were calculated using 100 replicates. The ML tree was visualised using FigTree v1.4.2, and the Bayesian analysis was performed using 40 million Markov Chain Monte Carlo (MCMC) generations, sampling a tree every 2000 generations. The convergence of the runs was verified using Tracer v1.7.1^51^. After removing the first 10% of the sampled 20,000 trees as burn-in, Bayesian posterior probabilities were calculated from the remaining trees using Tree Annotator v2.4.3^52^. FigTree v1.4.2 was used to illustrate the Bayesian tree. The Published D-loop sequences (GenBank) of another *Gallus* species (*G. varius*) were used as outgroups. Divergence times of haplotypes that were identified in the present study were estimated using BEAST v2^53^, in which the time of the root node of the tree was set at 10,000 years ago. To determine the fine-scale genetic relationships among the haplotypes detected in the present study and the 420 D-loop haplotypes obtained from the GenBank, median-joining network trees were reconstructed using PopART v1.7.2^54,55^.

### Network analysis of mtDNA D-loop haplotypes of red junglefowl in Asia

Phylogenetic relationships among D-loop haplotypes of red junglefowl were examined according to the haplotype network tree constructed using Popart v1.7.2.^54,55^ with 279 D-loop sequences from the present study and 221 D-loop sequences from previous studies (Supplementary Table S7) assessing *G. g. gallus, G. g. spadiceus, G. g. murghi,* and *G. g. bankiva* from nine countries. In total, 49, 7, 22, and 53 individuals of *G*. *g*. *murghi*, *G*. *g*. *bankiva*, *G*. *g*. *gallus*, and *G*. *g*. *spadiceus*, respectively, were assessed. The other 90 individuals whose subspecies names were unknown were referred as *G. gallus*.

### Analysis of genetic diversity and genetic structures based on mtDNA D-loop sequences

The nucleotide diversity (*pi*)^56^, number of haplotypes (*H*), and Watterson estimator per sequence (*Theta-w*)^57^ were calculated using DnaSP v5^58^. The demographic history of each population was estimated using Tajima’s *D* test^59^ implemented in DnaSP v5. Genetic distances between populations were assessed using Molecular Evolutionary Genetics Analysis version 10 (MEGA X)^60^ using two different indices: (1) the average number of nucleotide substitutions per site (*Dxy*); and (2) the number of net nucleotide substitutions per site between populations (*Da*).

### Analysis of genetic diversity using microsatellite DNA markers

Genetic diversity indices, namely AR, number of alleles per population (*Na*), polymorphic information content (*PIC*), null allele frequency (*NAF*), and *F* statistics (*F*_*IS*_, *F*_*ST*_, and *F*_*IT*_), were calculated for each of the microsatellite DNA markers, using MICROSATELLITE ANALYSER v4.05^61^ (*AR*, *F*_*IS*_, *F*_*ST*_, and *F*_*IT*_), GenAlEx v6.5^62^ (*Na*), and Cervus v3.0.7^63,64^ (*PIC* and *NAF*) (Supplementary Table S3). Subsequently, the genetic diversity indices were examined for each of the 10 indigenous chicken breeds and 12 populations of red junglefowl using MICROSATELLITE ANALYSER 4.05 for *AR*, GenAlEx 6.5 for *MNA* and the mean number of effective alleles (*Ne*, the number of alleles weighted by the square of the allele frequency), and Arlequin v3.5.2.2^65^ for the observed heterozygosity (*Ho*) and the expected heterozygosity (*He*) (Table 3). Null allele frequencies for each locus and population were estimated based on the expectation maximization algorithm^66^ using FreeNA^67^. The Chi-square test was used to test for Hardy–Weinberg equilibrium at each locus and in each population using Arlequin v3.5.2.2. Pairwise *Fst* and *Rst* genetic distances between populations were calculated using Arlequin v3.5.2.2. The *p* value was estimated by 10,000 permutation tests for calculating each pairwise genetic distance. Genetic relationships between all individual pairs were assessed by Nei’s angular genetic distance based on allele frequencies (*Da*)^68^ and the genetic distance based on the proportion of shared alleles (*Dps*)^69^, both of which were calculated using MICROSATELLITE ANALYSER v4.05.

Bayesian clustering analysis was performed to infer the number of genetic clusters for 10 indigenous chicken breeds and 12 red junglefowl populations using STRUCTURE v2.3^70^. The log probability values from K = 2 to K = 23 were estimated for a sampling period of 500,000 MCMC generations after a burn-in period of 500,000 generations under the admixture model and the correlated allele frequency model^71^. Twenty independent MCMC runs were performed for each K. Of the 20 MCMC runs for each K, those with variances of log likelihood more than twice those of the other MCMC runs were excluded from subsequent analyses. The clustering patterns of the remaining runs were analysed to generate one major clustering pattern in each K using CLUMPAK^72^. Afterward, the optimal K value was determined using the Evanno method^73^ in STRUCTURE HARVESTER v0.6.94^74^. To facilitate better understanding of the genetic differences between the two subspecies of red junglefowl, AMOVA was performed by separating 12 red jungle fowl populations into *G. g. gallus* and *G. g. spadiceus* using Arlequin v3.5.2.2.

## Supplementary Information

Supplementary Figure Legends.

Supplementary Figure S1.

Supplementary Figure S2.

Supplementary Figure S3.

Supplementary Figure S4.

Supplementary Tables.

## Data Availability

The DNA sequence data have been deposited in the DNA Data Bank of Japan, and all data are provided in the main text or as supplementary data.
