# Supplementary material for: Origin and evolutionary history of domestic chickens inferred from a large population study of Thai red junglefowl and indigenous chickens
Source: Sci Rep. 2021 Jan 21;11:2035. doi: 10.1038/s41598-021-81589-7 (PMC7820500; doi:10.1038/s41598-021-81589-7)
Supplement: Supplementary file 1 — Supplementary Figure Legends. [file 41598_2021_81589_MOESM1_ESM.pdf]

Origin and evolutionary history of domestic chickens inferred from a large population study of Thai red junglefowl and indigenous chickens

Authors: Ayano Hata[1,2,3], Mitsuo Nunome[4], Thanathip Suwanasopee[3], Prateep Duengkae[5], Soontorn Chaiwatana[6], Wiyada Chamchumroon[6],Takayuki Suzuki[1,4], Skorn Koonawootrittriron[3*], Yoichi Matsuda[1,4*] and Kornsorn Srikulnath[2,5*]

[1]Laboratory of Avian Bioscience, Department of Animal Sciences, Graduate School of Bioagricultural Sciences, Nagoya University, Nagoya, Aichi 464-8601, Japan

[2]Laboratory of Animal Cytogenetics and Comparative Genomics (ACCG), Department of Genetics, Faculty of Science, Kasetsart University, Chatuchak, Bangkok 10900, Thailand

[3]Tropical Animal Genetic Unit (TAGU), Department of Animal Science, Faculty of Agriculture, Kasetsart University, Chatuchak, Bangkok 10900, Thailand

[4]Avian Bioscience Research Center, Graduate School of Bioagricultural Sciences, Nagoya University, Nagoya Aichi 464-8601, Japan

[5]Special Research Unit for Wildlife Genomics, Department of Forest Biology, Faculty of Forestry, Kasetsart University, Chatuchak, Bangkok 10900, Thailand

[6]Department of National Parks, Wildlife and Plant Conservation, Chatuchak, Bangkok 10900, Thailand

*Corresponding and requests for materials should be addressed to Kornsorn Srikulnath (email: kornsorn.s@ku.ac.th), Skorn Koonawootrittriron (agrskk@ku.ac.th), and Yoichi Matsuda (yoimatsu@agr.nagoya-u.ac.jp)

Supplementary figures

Fig. S1. Bayesian phylogenetic tree of mtDNA D-loop sequence haplotypes that were found in 10 indigenous chicken breeds and 12 red junglefowl populations in Thailand. The tree was constructed with 420 D-loop sequences (A01, A02, …Y, and Z), which were defined by Miao *et al.*[5] and 22 D-loop sequences from GenBank (Table S6). The groups of haplotypes are shown at the right side of the tree: A, B, C, D (ZY), E, F, G, J and a minor-type of haplogoups H, I, K, X, and W, which is a complex of rare haplotypes, including Hap_05. The divergence time of each node is shown at the right side of the node. The nodes that have Bayesian posterior probabilities greater than 0.97 are indicated by magenta arrowheads. The haplotypes obtained in the present study are highlighted in orange. The phylogenetic position of a unique haplotype Hap_38 is shown by a blue arrowhead.

Fig. S2. Maximum-likelihood phylogenetic tree of mtDNA D-loop sequence haplotypes that were found in 10 indigenous chicken breeds and 12 red junglefowl populations in Thailand. The tree was constructed with 420 D-loop sequences (A01, A02, …Y, and Z), which were defined by Miao *et al.*[5] and 22 D-loop sequences from GenBank (Table S6). Groups of haplotypes are shown at the right side of the tree: A, B, C, D (ZY), E, F, G, J and a complex of rare haplogroups H, I, K, X, and W. The nodes that have bootstrap values greater than 50% are indicated by magenta arrow heads. The haplotypes obtained in the present study are highlighted in orange. The phylogenetic position of a unique haplotype Hap_38 is shown by a blue arrowhead.

Fig. S3. Neighbour-joining tree of 10 indigenous chicken breeds and 12 red junglefowl populations in Thailand, **constructed using microsatellite markers.** The tree was constructed using Nei's genetic distance based on the allele frequencies (*Da*) of 28 microsatellite markers. Ggg and Ggs within parentheses after the location names of red junglefowls indicate *Gallus gallus gallus* and *G. g. spadiceus*, respectively.

Fig. S4. Locations of red junglefowl sample collection in Thailand. The photographs of one individual at each location are shown except for two centres, Si Sa Ket and Petchaburi.
