# Supplementary figures and images for: Origin and evolutionary history of domestic chickens inferred from a large population study of Thai red junglefowl and indigenous chickens
Source: Sci Rep. 2021 Jan 21;11:2035. doi: 10.1038/s41598-021-81589-7 (PMC7820500; doi:10.1038/s41598-021-81589-7)

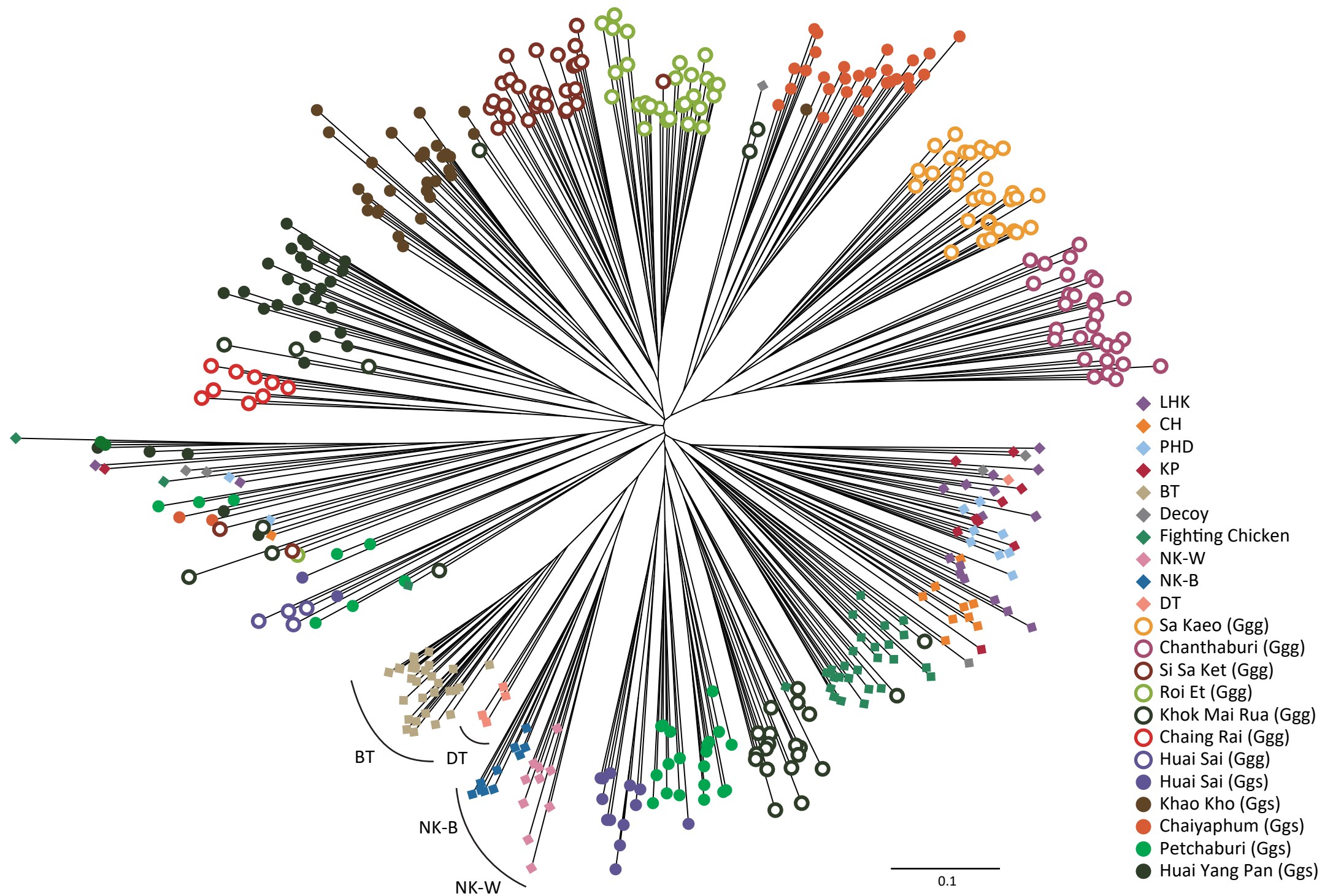

Fig. S3

Supplement: Supplementary file 4 — Supplementary Figure S3. [file 41598_2021_81589_MOESM4_ESM.pdf]

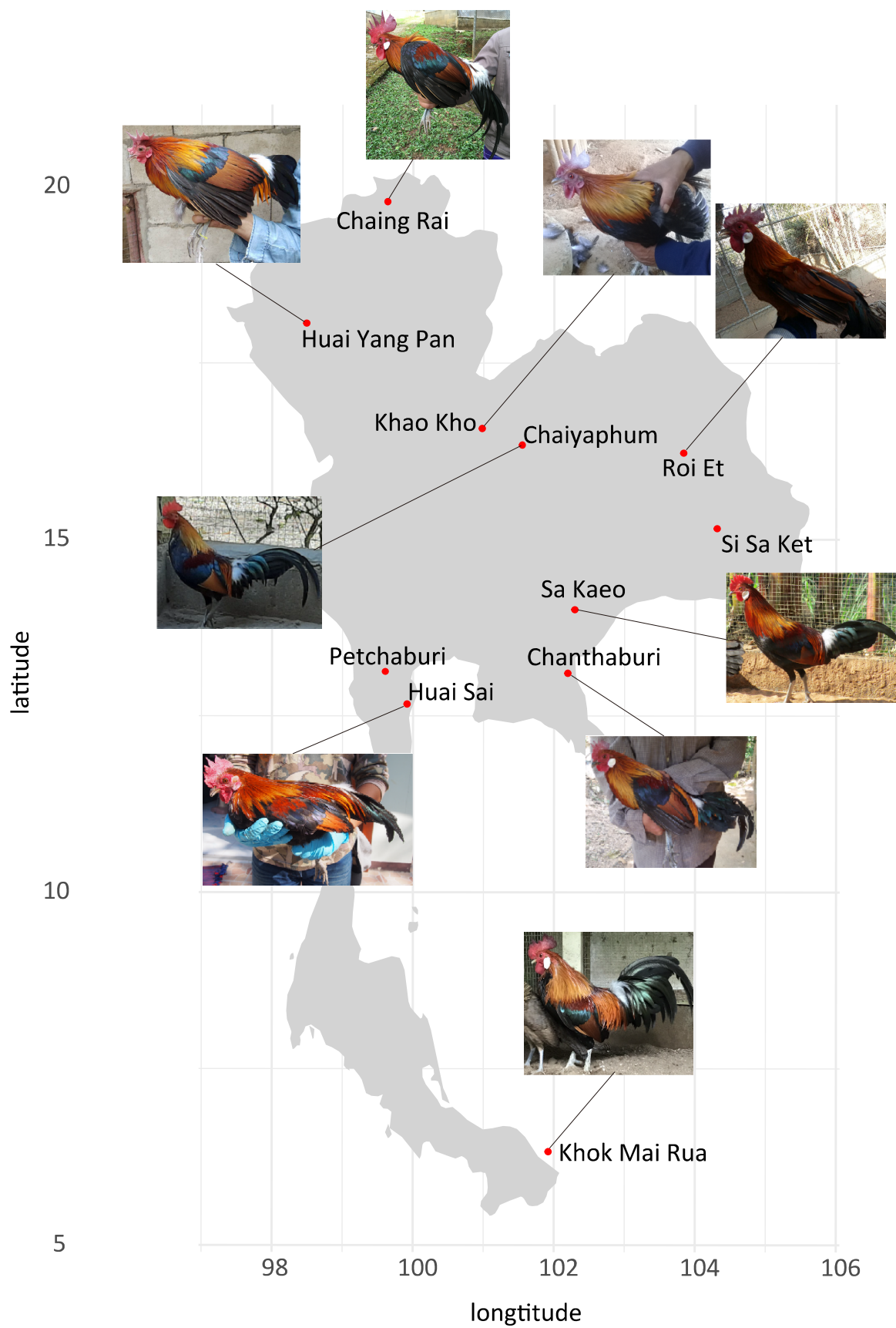

Fig. S4

Supplement: Supplementary file 5 — Supplementary Figure S4. [file 41598_2021_81589_MOESM5_ESM.pdf]
